# Perceived Racial Discrimination Is Associated with Greater Dietary Inflammatory Potential in Children and Adolescents in the Adolescent Brain and Cognitive Development Study

**DOI:** 10.1016/j.cdnut.2026.109539

**Published:** 2026-09-19

**Authors:** Penias Tembo, Travis R Moore, Olivia Affuso, Adolfo G Cuevas, Robert Aidoo, Michael D Wirth, James R Hébert, Danielle M Krobath

**Affiliations:** 1 Department of Epidemiology and Biostatistics, Arnold School of Public Health, University of South Carolina, Columbia, SC, United States; 2 Cancer Prevention and Control Program, Arnold School of Public Health, University of South Carolina, Columbia, SC, United States; 3 Department of Epidemiology, School of Public Health, University of Pittsburgh, PA, United States; 4 Council on Black Health, Knightdale, NC, United States; 5 Department of Social and Behavioral Sciences, New York University School of Global Public Health, New York, NY, United States; 7 Department is Biobehavioral Health and Nursing Science, College of Nursing, University of South Carolina, Columbia, SC, United States

**Keywords:** perceived racial discrimination, dietary inflammatory potential, diet quality, Adolescent Brain Cognitive Development (ABCD) Study

## Abstract

**Background:**

Perceived racial discrimination is a psychosocial stressor linked to chronic inflammation and diet-related disease risk among adults. Less is known about its effects among youth. Discrimination may influence youth chronic disease risk through dietary patterns with greater inflammatory potential, a modifiable pathway across the life course.

**Objectives:**

This study aims to examine the association between self-reported perceived racial discrimination and dietary inflammatory potential in a large, racially and sociodemographically diverse sample of youth in the United States.

**Methods:**

We analyzed longitudinal data from 6418 participants aged 9–12 y in the Adolescent Brain Cognitive Development Study. Perceived racial discrimination, assessed at time 1 (2018–2020) using the Youth Discrimination Measure, was modeled as a binary variable derived from a composite score, distinguishing youth reporting no discrimination from those reporting any discrimination. Children’s Dietary Inflammatory Index (C-DII) scores, quantifying the inflammatory potential of dietary patterns, were derived from parent-reported 7-d food frequency questionnaires at time 1 and time 2 (2019–2021). Time 2 C-DII scores were categorized into sample-derived quartiles: Q1 reflected the lowest and Q4 reflected the highest dietary inflammatory potential. Ordinal logistic regression assessed whether time 1 discrimination predicted higher time 2 C-DII quartile, adjusting for time 1 C-DII and sociodemographic covariates.

**Results:**

At time 1 (*n* = 6418), participants had a mean age of 11.97 y (SD = 0.64); 52.2% were male, and 60.5% were non-Hispanic White. Youth reporting racial discrimination at time 1 had 14% higher adjusted odds of being in a more proinflammatory C-DII quartile at time 2 than youth reporting no discrimination (odds ratio = 1.14; 95% confidence interval: 1.02, 1.26; *P* = 0.02).

**Conclusions:**

Perceived racial discrimination is associated with more proinflammatory dietary patterns during early adolescence. Future studies should evaluate whether dietary patterns mediate the association between discrimination and subsequent diet-related chronic disease risks in youth.

## Introduction

Poor diet is a significant contributor to the rapidly rising rates of preventable illness among children and adolescents in the United States [1,2], partially through its role in driving chronic, low-grade systemic inflammation [3]. According to recent estimates, if the United States continues on its current trajectory, rates of chronic diseases once considered a disease of adulthood, such as type 2 diabetes, are projected to increase by as much as 673% by the year 2060 [4]. These increases are expected to disproportionately affect racially minoritized youth. For example, projections indicate that by 2060, type 2 diabetes prevalence among non-Hispanic Black youth could rise by >901%, compared with an increase of ∼142% among non-Hispanic Whites [4]. Therfore, mitigating the pervasive effects of racial discrimination on chronic disease risk factors, such as proinflammatory dietary patterns, represents a potential step toward improving the overall health and nutritional status of future generations.

Perceived racial discrimination, often referred to as personally mediated racism, describes an individual’s perception of being treated unfairly or negatively because of their race or ethnicity [5]. Racial discrimination is increasingly recognized as a major psychosocial determinant of health behaviors and chronic disease among adults. However, less attention has been directed toward child and adolescent populations [6,7]. A recent analysis of data collected between 2017 and 2019 from a United States cohort of 10-y and 11-y olds revealed that 4.8% of participants reported being treated unfairly because of their race, ethnicity, or skin color [8]. Moreover, significant racial disparities emerged: only 2.8% of White children reported experiencing racial discrimination, compared with 10.0% of Black children, 5.4% of Hispanic children, and 6.2% of Asian children [8].

Early exposure to discrimination is concerning, given that children and adolescents are in critical stages of identity formation, social learning, and physiological growth [9]. As a chronic stressor, racial discrimination has been associated with adverse long-term health outcomes. Cuevas et al. [10] found that adults who reported experiencing more racial discrimination during childhood and adolescence were more likely to develop cardiovascular-related health problems later in life, conditions in which chronic, low-grade inflammation plays a central role [11]. Although the mediating pathways are complex and not yet fully elucidated, exposure to race-related stressors during childhood and adolescence may instigate psychosocial maladaptation and unhealthy lifestyle behaviors, contributing to health disparities that persist into adulthood [9,12,13].

Most prior studies linking racial discrimination with diet have examined intake of individual foods or nutrients rather than overall dietary patterns [14]. For example, Ehrlich et al. [15] found that experiences of discrimination were associated with higher snack calorie consumption among preadolescents, particularly for individuals with overweight or obesity. Although useful for describing discrete behaviors, single nutrient and single food approaches do not capture the holistic effects of diet on biological processes [16]. In contrast, dietary pattern analyses provide a more comprehensive assessment of diet as a complex exposure, making it possible to evaluate how overall eating behaviors influence biological pathways such as inflammation, a substrate that has been implicated in mechanisms driving the etiology of numerous chronic diseases [17]. Therefore, to quantify the inflammatory potential of diet, the Dietary Inflammatory Index (DII) was created to categorize dietary patterns along a continuous spectrum, ranging from diets that reduce inflammation (anti-inflammatory) to those that increase inflammation (proinflammatory) [18,19]. Because children’s dietary patterns differ from those of adults and they have unique nutrient requirements, a version specifically designed for younger populations, the Children’s DII (C-DII) [20], was developed and validated against markers of systemic inflammation and oxidative stress [21].

To our knowledge, no prior study has examined whether perceived racial discrimination is associated with elevated dietary inflammatory potential in adolescents. This gap is particularly salient given evidence of elevated nondietary inflammation among some racially minoritized youth [22]. This information may have crucial implications for addressing racial health disparities, particularly because many minoritized racial groups in the United States are already known to experience elevated levels of stress-induced inflammation [22]. The C-DII offers the unique opportunity to quantify links between racial discrimination and the inflammatory potential of the diet consumed by this population. This study aimed to measure the association between youth-reported racial discrimination and dietary inflammatory potential in a large, racially and sociodemographically diverse sample of youth in the United States. We hypothesized that perceived racial discrimination at Time 1 would be associated with higher C-DII scores at Time 2, approximately one year later.

## Methods

### Study design

The Adolescent Brain and Cognitive Development (ABCD) Study is a large, multisite cohort of 11,876 children aged 9–10 y at baseline, enrolled between 1 September, 2016, and 31 August, 2018, across 21 sites in the United States [23]. The cohort at baseline was ∼47.8% female, 52.1% White, 15.0% Black, 20.3% Hispanic, and 10.5% other race. Recruitment procedures have been described previously, with Institutional Review Board approval obtained from the University of California, San Diego, and all 21 participating study sites [24].

### Exposure variable

Perceived racial discrimination was assessed in ABCD at year 2 follow-up (2018–2020), herein referred to as “Time 1,” using the Youth Discrimination Measure (YDM). The YDM is a well-validated scale for use in adolescent populations with strong predictive validity for stress-related mental health outcomes among racially diverse adolescents in the ABCD cohort [25,26]. The overarching focus of the YDM is on adolescent self-reported experiences of racial discrimination. YDM assesses whether in the previous year (yes/no) youth experienced unfair treatment in their day-to-day lives owing to their race or ethnicity. Additional scale questions assess if discrimination was perpetrated by peers, teachers, or other adults using a scale that employs 5 frequency response codes (1 = never, 2 = rarely, 3 = sometimes, 4 = often, 5 = very often). With the same frequency scale, respondents are asked to report on a series of questions related to perceived belonging in American society. In this study, binary responses were averaged with frequency items to form a mean everyday discrimination score, with higher values indicating greater discrimination exposure. To address 0 inflation in the data and in a manner similar to what has been done previously [27], we then created a binary variable by which individuals with a mean score of 1.0 (indicating “Never” across all discrimination items) were classified as not experiencing discrimination, whereas those with a mean score greater than 1.0 were categorized as having experienced racial discrimination.

### Outcome variable

To construct the primary outcome variable, the C-DII score at ABCD year 3 follow-up (2019–2021), herein referred to as “Time 2,” we used dietary intake data collected via the Block Kids Food Screener (BKFS). The BKFS is a validated 41-item Food Frequency Questionnaire (FFQ) [28] that was administered to parents at Time 1 and Time 2. In the BKFS, parents reported how often and how much their child consumed various foods and beverages in the previous 7 d. Summary data from the BKFS were provided by NutritionQuest (Berkeley Analytics, Inc., Berkeley, CA, USA), and the following components were used to calculate the C-DII score: caffeine, carbohydrate, total fat, fiber, MUFA, PUFA, saturated fat, cholesterol, protein, iron, magnesium, niacin, riboflavin, thiamin, vitamin B6, vitamin C, vitamin E, zinc, β-carotene, folic acid, selenium, vitamin A, and vitamin B12, along with total energy. Thereafter, we computed the C-DII, a previously validated measure of the inflammatory potential of a child’s diet [20]. The C-DII has been validated against markers of inflammation, immune function, and oxidative stress [20]. Briefly, the C-DII was calculated by deriving a *Z*-score for each nutrient, based on comparison of the child’s reported intake to a global reference mean and SD. These *z*-scores were converted to proportions, multiplied by 2, and centered on a scale from −1 to +1. Each centered value was then multiplied by the corresponding literature-based inflammatory effect score for that nutrient, and values were summed to yield the overall C-DII score. C-DII scores can range from strongly negative to strongly positive [20]. Negative scores reflect more anti-inflammatory diets (e.g., diets higher in fruits, vegetables, whole grains, and other nutrient-dense foods), whereas positive scores reflect more proinflammatory diets (e.g., diets higher in saturated fat, added sugars, and processed foods). In this analysis, Time 2 C-DII scores were categorized into quartiles (Q1–Q4) based on their distribution in the sample: Q1 (≤ −4.44, −1.25), Q2 (−1.24 to − 0.15), Q3 (−0.14 to −0.87), and Q4 (0.88 to 4.45). Lower quartiles represent diets with the least inflammatory potential, whereas higher quartiles represent diets with the greatest inflammatory potential.

### Covariates

Sociodemographic data were collected from parents/caregivers, who reported the child’s age (continuous: in years), sex assigned at birth (binary: male or female), racial/ethnic identity [categorical: White, Black, Hispanic/Latino (any race), other race (which includes Asian or Native Hawaiian or Pacific Islander, American Indian or Alaska Native, multiracial, or other race)], nativity (binary: United States born or non-United States born), total household income (categorical: <$25,000, $25,000–$49,999, $50,000–$74,999, $75,000–$99,999, $100,000–$199,999, and ≥$200,000), and parent/caregiver maximum education level (categorical: high school diploma or less, some college, a Bachelor’s or Associate’s degree, and a graduate or professional degree). Sociodemographic covariates were collected at baseline (2016–2018.)

### Statistical analysis

To describe the demographic characteristics of the study sample, continuous variables were summarized by mean and SD, and categorical variables by count and percentage (%). Descriptive statistics were summarized overall and stratified by perceived racial discrimination status (no discrimination compared with some discrimination). To examine the association between perceived racial discrimination at Time 1 and dietary inflammatory potential (via C-DII quartiles) at Time 2, we fit an ordinal logistic regression model with perceived racial discrimination modeled as a binary exposure. Covariates included age, sex assigned at birth, race/ethnicity, household income, parental education, child’s nativity, and the mean C-DII score at Time 1. These variables were selected *a priori* based on theoretical and empirical evidence linking sociodemographic factors to both interpersonal discrimination and dietary behaviors. Sex assigned at birth was included because dietary patterns and discriminatory experiences may vary by sex [29]. Age was included because youth exposure to and recognition of racial discrimination can change as they enter adolescence [27], whereas diet quality tends to decline during this period [30]. We included the child’s racial/ethnic identity as a marker of social position that reflects how children are racialized in the United States and, consequently, differences in their likelihood of experiencing perceived racial discrimination [31]. Household income and parental education were included as indicators of socioeconomic position that shape both exposure to discrimination and access to the resources supportive of a healthy diet [32]. Finally, nativity status was included as a marker of immigration-related social context because prior research has connected nativity and acculturation to differences in diet quality among United States youth [33,34]. The proportional odds assumption was tested using the gologit2 command with the autofit option in Stata. Results supported the use of the ordinal logistic model, as the proportional odds assumption held across all covariates. In exploratory analyses, we tested for interactions between perceived racial discrimination and age, sex, race/ethnicity, and parental education in separate adjusted models because these characteristics represent key, theory-driven axes along which both exposure to racial discrimination and dietary responses may differ. Unadjusted and adjusted odds ratios (ORs and aORs, respectively) with corresponding 95% confidence intervals (CIs). Statistical significance was defined as a 2-sided *P < 0.05*. We conducted a complete case analysis by excluding participants with missing data for any of the analytical variables of interest. Data analyses were conducted from October 2024 to June 2025 using Stata SE version 17 (StataCorp LLC, College Station, TX, USA).

## Results

Table 1 presents the characteristics of the 6418 participants in ABCD cohort included in this study at Time 1, overall and stratified by discrimination status. In total, 27.1% (*n* = 1741) of youth reported experiencing racial discrimination. The mean age was 11.97 y (SD = 0.64). Overall, 52.2% (N = 3350) of the sample were male, and 60.5% (N = 3885) were identified by parents/caregivers as non-Hispanic White. The prevalence of self-reported discrimination differed by race/ethnicity (*P* < 0.001). Among youth who reported experiencing discrimination, non-Hispanic Black adolescents comprised a larger proportion of the discrimination-exposed group (15.9%; N = 277) compared with their representation in the overall sample (9.8%; N=631). Hispanic/Latino youth similarly accounted for a greater share of those reporting discrimination (23.4%; N = 408) relative to their proportion in the full sample (18.0%; N = 1157). In contrast, White adolescents comprised a smaller proportion of the discrimination-exposed group (46.3%; N=806) than of the overall sample (60.5%; N = 3885). Furthermore, socioeconomic characteristics differed significantly by discrimination status (*P* < 0.001). Youth from lower-income households comprised a larger share of those reporting racial discrimination. For example, adolescents from households earning <$25,000 annually accounted for 16.1% (N = 280) of the discrimination-exposed group, compared with 8.4% (N = 393) of youth reporting no discrimination. In contrast, youth from households earning $200,000 or more represented a smaller proportion of the discrimination group (9.3%; N = 161) relative to the nondiscrimination group (13.8%; N = 645). Similar patterns were observed for parental education. Adolescents whose parents had a high school education or less constituted a greater proportion of the discrimination group (15.9%; N = 277) compared with the nondiscrimination group (9.6%; N = 449). Youth whose parents held graduate degrees represented a smaller share of those reporting discrimination (23.2%; N = 404) than of those reporting no discrimination (30.6%; N = 1433) (*P* < 0.001). Across the full sample, the mean C-DII score was slightly higher at Time 2 (*β* = −0.19; SD = 1.44) than at Time 1 (*β* = −0.27; SD=1.45), indicating a modest shift toward more proinflammatory dietary patterns as youth get older. However, those who reported discrimination demonstrated higher mean C-DII scores (i.e., more proinflammatory) at both time points compared with those who did not report discrimination (Time 1: discrimination *β* = −0.16; SD = 1.43, −0.09 compared with nondiscrimination *β* = −0.31; SD = 1.45; Time 2: discrimination *β* = −0.04; SD = 1.43, compared with nondiscrimination *β* = −0.26; SD = 1.44; *P* < 0.001 for both). Finally, the distribution of Time 2 C-DII quartiles differed significantly by discrimination status (*P* < 0.001). Among youth reporting racial discrimination, a greater proportion were classified in the most proinflammatory diet category (Q4; 29.0%; N = 505) than in the most anti-inflammatory category (Q1; 21.2%; N = 369).

**TABLE 1:** Sociodemographic characteristics of the Adolescent Brain and Cognitive Development at time 1, overall and by self-reported racial discrimination exposure

Characteristics	(A) Full sample *n* = 6418	(B) Youth reporting no discrimination (*n* = 4677)	(C) Youth reporting discrimination (*n* = 1741)	*P* value
Age (y) mean (SD)^1^	11.97 (0.64)	11.95 (0.65)	12.02 (0.65)	<0.001
Sex^2^				
Female	3068 (47.80)	2272 (48.58)	796 (45.72)	0.04
Male	3350 (52.20)	2405 (51.42)	945 (54.28)	
Race/ethnicity^2^				
Black	631 (9.83)	354 (7.57)	277 (15.91)	<0.001
Hispanic/Latino	1157 (18.03)	749 (16.01)	408 (23.43)	
Other race	745 (11.61)	495 (10.58)	250 (14.36)	
White	3885 (60.53)	3079 (65.83)	806 (46.30)	
Nativity^2^				
Non-United States born	169 (2.63)	112 (2.39)	57 (3.27)	0.05
United States born	6249 (97.37)	4565 (97.61)	1684 (96.73)	
Parent education^2^				
High school or less	726 (11.31)	449 (9.60)	277 (15.91)	<0.001
Some college	976 (15.21)	633 (13.53)	343 (19.70)	
Associate’s/bachelor’s	2879 (44.86)	2162 (46.23)	717 (41.18)	
Graduate degree	1837 (28.62)	1433 (30.64)	404 (23.21)	
Household income^2^				
<$25,000	673 (10.49)	393 (8.40)	280 (16.08)	<0.001
$25,000–$49,999	893 (13.91)	569 (12.17)	324 (18.61)	
$50,000–$74,999	906 (14.12)	639 (13.66)	267 (15.34)	
$75,000–$99,999	1016 (15.83)	760 (16.25)	256 (14.70)	
$100,000–$199,999	2124 (33.09)	1671 (35.73)	453 (26.02)	
≥$200,000	806 (12.56)	645 (13.79)	161 (9.25)	
C-DII score at time 1^1^^,^^3^	–0.27 (1.45)	–0.31 (1.45)	–0.16 (1.43)	<0.001
C-DII score at time 2^1^^,^^4^	–0.19 (1.44)	–0.26 (1.44)	–0.04 (1.43)	<0.001
Time 2 C-DII quartile^2^^,^^5^				
Quartile 1	1605	1236 (26.43)	369 (21.19)	<0.001
Quartile 2	1604	1184 (25.32)	420 (24.12)	
Quartile 3	1605	1158 (24.76)	447 (25.67)	
Quartile 4	1604	1099 (23.50)	505 (29.01)	

*Abbreviation*: C-DII, Children’s Dietary Inflammatory Index.

1 Continuous variables are summarized by mean and SD and compared using 2 sample *t* tests.

2 Categorical variables are summarized by count and percentage [*n* (%)] and compared using χ^2^ tests.

3 Range of C-DII score at time 1: −4.37 to 3.64.

4 Range of C-DII score at time 2: −4.44 to 4.45.

5 C-DII quartiles (based on the sample distribution) were: Q1 = −4.44 to −1.25, Q2 = −1.24 to −0.15, Q3 = −0.14 to 0.87, and Q4 = 0.88 to 4.45; higher quartiles indicate a more proinflammatory diet.

The full results of the ordinal logistic regression analyses are presented in Table 2. The global Wald test indicated no violation of the proportional odds assumption (*χ*^2^(34) = 22.78; *P* = 0.93), supporting the use of the ordinal logistic regression model. In the adjusted ordinal logistic regression model, adolescents who reported discrimination exposure had 14% higher odds of being in a more proinflammatory diet category (aOR = 1.14; 95% CI: 1.02, 1.26; *P* = 0.02) compared with those who did not. Males had significantly higher odds of consuming a more proinflammatory diet than females (aOR = 1.39; 95% CI: 1.27, 1.53; *P* < 0.001). Compared with White adolescents, those identifying as Hispanic/Latino had significantly lower odds of proinflammatory dietary patterns (aOR = 0.86; 95% CI: 0.75, 0.99; *P* = 0.03), whereas significant differences for Black youth and those in the other race category were not detected. Having a parent with a graduate degree was associated with lower odds of being in a more proinflammatory diet quartile (aOR = 0.79; 95% CI: 0.65, 0.96; *P* = 0.02). Higher C-DII scores at Time 1 were significantly associated with greater odds of being in a more pro-inflammatory dietary quartile at Time 2 (aOR = 2.38; 95% CI: 2.28, 2.47; *P* < 0.001). There was no evidence of interaction between perceived racial discrimination and age, sex, race/ethnicity, or parental education (nonsignificant models not shown).

**TABLE 2:** Ordinal logistic regression results examining the association between time 1 perceived racial discrimination exposure and time 2 C-DII Quartiles

Predictor variables	Unadjusted OR			Adjusted OR		
	C-DII quartile	*P* value	95% CI	C-DII quartile	*P* value	95% CI
Discrimination category						
No discrimination	Ref	—	—	Ref	—	—
Some discrimination	1.32	<0.001	1.19, 1.45	1.14	0.02	1.02, 1.26
Age	—	—	—	1.00	0.18	0.99, 1.01
Sex	—	—	—			
Female	—	—	—	Ref	—	—
Male	—	—	—	1.39	<0.001	1.27, 1.53
Race/ethnicity	—	—	—			
White	—	—	—	Ref	—	—
Black	—	—	—	1.14	0.14	0.96, 1.36
Hispanic/Latino	—	—	—	0.86	0.03	0.75, 0.99
Other race	—	—	—	1.04	0.65	0.89, 1.20
Household income	—	—	—			
<$25,000	—	—	—	Ref	—	—
$25,000–$49,999	—	—	—	1.06	0.57	0.87, 1.28
$50,000–$74,999	—	—	—	1.02	0.81	0.83, 1.25
$75,000–$99,999	—	—	—	0.87	0.17	0.71, 1.07
$100,000–$199,999	—	—	—	0.84	0.09	0.69, 1.02
≥$200,000	—	—	—	0.88	0.26	0.70, 1.10
Parent education	—	—	—			
High school or less	—	—	—	Ref	—	—
Some college	—	—	—	0.92	0.49	0.77,1.13
Associate’s/bachelor’s	—	—	—	0.82	0.08	0.72,1.02
Graduate degree	—	—	—	0.79	0.02	0.65, 0.96
Nativity	—	—	—			
United States born	—	—	—	Ref	—	—
Non-United States born	—	—	—	0.82	0.21	0.61, 1.12
Mean C-DII score at time 1	—	—	—	2.38	<0.001	2.28, 2.47

Data shown are results of an ordinal logistic regression model estimating the association of youth-reported interpersonal racial discrimination at time 1 (year 2 follow-up) with odds of being in a higher C-DII quartile (more proinflammatory diet) at time 2 (year 3 follow-up). Unadjusted and adjusted ORs, 2-sided *P* values, and 95% CIs are shown. Reference categories are indicated in the table. Adjusted models accounted for the mean C-DII score at time 1, child age, child sex assigned at birth, child race/ethnicity, household income, parental education, and child nativity status.

Abbreviations: C-DII, Children’s Dietary Inflammatory Index; CI, confidence interval; OR, odds ratio.

## Discussion

This study provides longitudinal evidence that adolescents who report experiences of racial discrimination may have higher dietary inflammatory potential over time, even after adjustment for sociodemographic factors and baseline diet quality. Specifically, youth who reported experiencing racial discrimination had significantly higher odds of consuming a more proinflammatory diet compared with their peers who did not experience discrimination over one year. Time 1 C-DII score was the strongest predictor of the inflammatory potential of diet in adolescents over time, which is to be expected given that dietary patterns typically remain relatively stable over short periods. Overall, these results suggest that psychosocial stressors related to racial/ethnic identity may shape diet quality during early adolescence, a sensitive developmental period characterized by increasing autonomy over food choices and heightened vulnerability to stress [9,12,13].

### Interpretation in the context of prior literature

These results extend prior work linking racial discrimination to unhealthy eating behaviors among youth, including emotional eating, increased consumption of energy-dense foods, and lower intake of fruits and vegetables [15,35]. Most existing studies have relied on cross-sectional designs, small sample sizes, or intake of individual foods or nutrients, limiting inference about broader population-level dietary patterns [14]. For instance, Ehrlich et al. [15] observed that adolescents who reported higher levels of perceived racial discrimination consumed more snack foods and sugar-sweetened beverages over time, suggesting that discrimination-related stress may be associated with shifts toward more energy-dense dietary patterns during adolescence. By contrast, this study assessed the overall inflammatory potential of adolescents’ diets using the C-DII, which reflects diet as a biologically relevant outcome [12,13]. The longitudinal association observed in a large, diverse cohort over a 1-y period, while modest in magnitude, aligns with theoretical and empirical evidence suggesting that chronic psychosocial stressors may influence health-relevant behaviors across development [12,13].

While the present findings do not demonstrate mediation or causal pathways linking discrimination to chronic disease outcomes, they do suggest that greater inflammatory potential of the diet may represent a plausible, modifiable behavioral correlate of discrimination-related stress in youth. This interpretation is consistent with findings from Cuevas et al. (2023), who reported that perceived racial discrimination was associated with increases in BMI z-scores and waist circumference over one year among youth in the ABCD cohort, as well as broader evidence linking proinflammatory dietary patterns with cardiometabolic risk [36]. In addition, Heard-Garris et al. [36] found that Black individuals who reported higher levels of racial discrimination during adolescence and early adulthood were more likely to develop metabolic syndrome in early adulthood, even after accounting for socioeconomic factors. Although dietary pathways were not directly tested, the authors highlighted chronic psychosocial stress and stress-related behavioral responses as potential contributors to downstream cardiometabolic risk. Together, these findings support the plausibility of diet-related inflammation as one of several behavioral correlates of discrimination-related stress, while underscoring the need for future studies designed to test mediation and evaluate potential causal pathways [10,22].

### Situating interpersonal discrimination within broader food systems

Although experiences of perceived racial discrimination are measured at the interpersonal level, they occur within broader social and structural contexts that shape food exposure and dietary choice [37]. Discrimination is embedded within food systems through interconnected structural, institutional, and interpersonal processes that influence food availability, marketing, affordability, and norms [2,14,37]. For example, racially minoritized youth are disproportionately exposed to targeted marketing and are more likely to reside in environments with limited access to affordable, nutrient-dense foods [2,14]. Studies have shown that Black and Hispanic youth experience disproportionately high exposure to marketing of ultraprocessed and energy-dense foods across television, digital media, and neighborhood retail environments, independent of individual preferences [2,14]. These patterned exposures reflect broader policy dynamics and commercial determinants that shape food environments and may constrain dietary choice, particularly for youth already navigating discrimination-related stress. In this context, interpersonal experiences of racial discrimination may interact with structurally patterned food environments to influence dietary inflammatory potential, without placing undue responsibility on individuals for their dietary choices.

Framing food systems as systems of discrimination underscores how interpersonal experiences of racism intersect with policies, institutional practices, and commercial strategies that collectively shape dietary patterns and health inequities [37]. From this perspective, the association observed in this study should be understood as occurring within a wider constellation of social and policy environments that constrain dietary options and normalize exposure to proinflammatory foods for some populations. Adolescence may be a particularly consequential period for these dynamics, given developmental changes in stress physiology, identity formation, and eating autonomy [9]. Although this study cannot disentangle these multilevel processes, it paves the way for future studies and highlights the importance of situating individual-level experiences of discrimination within broader contexts that influence diet quality over time [12,22].

### Implications for research, practice, and policy

The findings of this study have several implications for research, practice, and policy. First, they suggest that experiences of racial discrimination should be considered alongside more commonly studied social determinants when examining diet quality and the inflammatory potential of diet in youth. Interventions that focus solely on individual dietary behavior change may be insufficient if they do not account for psychosocial stressors and the structural conditions that shape dietary exposure and potential coping responses [12,13,22].

Second, these results underscore the relevance of policy and environmental approaches that address both discrimination and inequities in food environments. Examples include policies that limit targeted marketing of ultraprocessed foods to children, strengthen nutrition standards in schools and community settings, and reduce structural barriers to accessing affordable, nutrient-dense foods in historically marginalized neighborhoods and communities [2,14]. Although this study did not evaluate such interventions, its findings support the importance of embedding nutrition strategies within broader equity-oriented policy frameworks [37].

Finally, this work highlights the need for future research that integrates systems-oriented and implementation-focused approaches to better understand how discrimination-related stress, food environments, and dietary behaviors interact across development [37]. Longitudinal and mixed-methods studies that attend to multilevel influences may help identify leverage points for intervention while avoiding overattribution of responsibility to individual choice. Such approaches are likely to be critical for informing equitable strategies to reduce the inflammatory potential of diet and downstream chronic disease risk among youth [10,17,22].

### Strengths and weaknesses

This study has several important strengths. First, our findings identify a novel potential behavioral mechanism through which experiences of racial discrimination may influence chronic disease risk, warranting further investigation. More, this large, racially and socioeconomically diverse cohort enhances the generalizability of our findings. Finally, the use of a validated youth self-reported measure of racial discrimination and a well-established measure of dietary inflammatory potential strengthens the internal validity of our study. Despite its strengths, the study has several limitations that warrant consideration. Dietary intake was reported by parents via an FFQ, which is subject to reporting bias and measurement error. Parents of children experiencing discrimination may differentially report their child’s intake, potentially influencing C-DII categorization. Additionally, the BKFS provides estimates of caloric intake, but it was designed primarily to assess food groups, added sugars, and certain key nutrients. Although the BKFS was not designed to comprehensively estimate total energy intake, it provided the dietary intake data necessary to calculate C-DII scores in the ABCD cohort. Thus, we were able to account for energy intake in this analysis by deriving C-DII scores. Nonetheless, residual confounding by total energy intake cannot be ruled out. It is also important to note that the DII and its derivatives (i.e., the C-DII and E-DII) were designed to capture overall dietary inflammatory potential rather than isolated nutrient effects. It has been validated and applied in pediatric cohorts where more reliable estimates of total energy intake are available [38]. Perceived discrimination was modeled as a binary exposure to account for zero inflation in responses, but this approach may have obscured dose–response associations between frequency and severity of discrimination and the inflammatory potential of diet. In addition, the 1-y follow-up interval was relatively short, and thus, longer term data are needed to determine whether observed differences in diet quality persist or intensify throughout adolescence. Despite adjustment for multiple sociodemographic covariates, residual and unmeasured confounding cannot be excluded. Finally, the 7-d FFQ captures usual dietary intake but not acute, stress-driven eating behaviors, which may also represent important pathways linking discrimination to more proinflammatory dietary patterns.

Overall, long-term solutions are needed to mitigate the effects of racial discrimination on diet quality by addressing overlapping multilevel factors, including food environments, and interpersonal social relationships [37]. In addition, future studies building on our work should examine dose-response relationships between the severity and type of perceived racial discrimination and dietary inflammatory potential, as well as the potential mediating role of pro-inflammatory diet patterns in the association between discrimination and chronic disease risk among marginalized adoloscent populations [39]. Intersectional analyses (e.g., by race, gender, and socioeconomic position) are needed to understand which groups may be most vulnerable to these effects. Future work should also be directed toward understanding the precise temporal association between racial discrimination and dietary responses. For example, diet may affect mood and stress responses directly, thus dampening the effect of external influences on stress [40,41]. Future research also should explore how these behavioral responses interact with underlying biological processes to influence long-term disease risk. Finally, integrating policy and environmental measures will be important for testing whether reducing exposure to discrimination and improving food environments can jointly mitigate diet-related inflammation and its long-term health consequences.

In conclusion, in a large, sociodemographically diverse cohort of United States adolescents, we found that experiences of racial discrimination were associated with more proinflammatory dietary patterns. These results suggest that psychosocial stressors, such as racial discrimination, may contribute to chronic disease outcomes not only through direct physiological stress pathways, but also by shaping diet quality during critical periods of growth. Our findings underscore the importance of addressing discrimination in youth as part of broader efforts to reduce racial health disparities, especially by highlighting the inflammatory potential of diet as an early, modifiable pathway. Interventions that target both interpersonal racism and structural barriers to healthy eating may help mitigate long-term risks for chronic disease.

## Author contributions

The authors’ responsibilities were as follows – PT, DMK: formulated hypotheses, conceptualized the study, designed the modeling strategy, and performed the analysis; PT, TRM, DMK: wrote the first draft of the manuscript; OA, AGC, MDW, JRH: assisted with interpreting the results; and all authors: reviewed and assisted with writing/revising the manuscript and read and approved the final manuscript.

## Data availability

The data used in this study were obtained from the Adolescent Brain Cognitive Development (ABCD) Study via the National Institute of Mental Health (NIMH) Data Archive (NDA). These data cannot be shared directly by the authors. Researchers may access ABCD data by completing the required Data Use Certification and Data Use Agreement through the NIMH NDA. Analytic code and variable codebooks will be made available to qualified ABCD data users upon request.

## Declaration of generative AI and AI-assisted technologies in the writing process

The author(s) declare that no generative AI or AI-assisted technologies were used in the writing of this manuscript.

## Funding

This work was supported by Washington University in St. Louis WU-CDTR (grant number P30DK092950 from the NIDDK). The content is solely the responsibility of the authors and does not represent the official views of the WU-CDTR or NIDDK. JRH and PT were supported by the US National Cancer Institute-funded REMEDY Study grant U01 CA272977-01, which is part of the MeDOC Consortium. The content is solely the responsibility of the authors and does not necessarily represent the official views of the National Institutes of Health.

## Conflict of interest

JRH owns a controlling interest in Connecting Health Innovations (CHI) LLC, a company that has licensed the right to his invention of the Dietary Inflammatory Index (DII) from the University of South Carolina to develop computer and smartphone applications for patient counseling and dietary intervention in clinical settings. CHI owns the E-DII and C-DII. The subject matter of this paper has no direct bearing on that work, nor has any CHI-related activity exerted any influence on this project. This did not and will not alter our adherence to the journal policies on sharing data and materials, but note that we are unable to share the computational algorithm, which is protected by trademark and patent law. All other authors declare no conflict of interest.
